# Cardiac rehabilitation for the transient ischaemic attack (TIA) and stroke population? Using the Medical Research Council (MRC) guidelines for developing complex health service interventions to develop home-based cardiac rehabilitation for TIA and ‘minor’ stroke patients

**DOI:** 10.1136/bjsports-2018-099593

**Published:** 2018-09-04

**Authors:** Neil Heron

**Affiliations:** 1 Department of General Practice and Primary Care, Queen’s University, Belfast, UK; 2 Centre for Public Health Research, Queen’s University, Belfast, UK; 3 Centre for Public Health, UKCRC Centre of Excellence for Public Health Research (NI), Belfast, UK

**Keywords:** rehabilitation, non-communicable disease, cardiovascular, prevention

## What did I do?

I developed a novel home-based rehabilitation programme, including ‘*The Healthy Brain Rehabilitation Manual*’, for patients with a first transient ischaemic attack (TIA) or ‘minor’ stroke of atherosclerotic origin, using the core components of home-based cardiac rehabilitation (CR) and conducted a pilot randomised controlled trial (RCT) to evaluate its effectiveness.

## Why did I do it?

CR is an effective form of secondary prevention for cardiovascular disease. CR after myocardial infarction results in reduced reinfarction risk and all-cause mortality.^1^ However, despite sharing similar pathology with coronary heart disease and the 90-day risk of further vascular events after a TIA or ‘minor’ stroke being as high as 18%,^2 3^ the value of CR for patients with a TIA or ‘minor’ stroke is unclear.

## How did I do it?

I followed the Medical Research Council guidelines for developing complex health service interventions. First, I conducted a systematic review (SR) of secondary prevention lifestyle interventions initiated within 90 days of a TIA or ‘minor’ stroke and then a SR on the use of behaviour change techniques (BCTs) in home-based CR. I used the SRs’ findings to adapt a home-based CR manual and design an intervention that was refined following stakeholder input (TIA/minor stroke patients and carers; clinical academics and health professionals). Then, to assess the applicability of the intervention, I conducted a feasibility study. Patients, recruited from hospital clinics within 4 weeks of a first TIA or minor stroke, were randomly allocated to three groups^1^: (1) standard/usual care^2^; (2) CR manual^3^; and (3) CR manual plus a pedometer. All groups received telephone follow-up 1 and 4 weeks postenrolment and were reviewed after 6 weeks.

Following the feasibility study and further intervention refinement, I conducted a 12-week pilot study to test the study protocol before a definitive RCT. Participants, recruited from four different centres, <4 weeks after their first TIA or ‘minor’ stroke, were randomly allocated to: (1) standard care (n=12); (2) CR manual, pedometer and general practitioner follow-up (n=14); and (3) CR manual, pedometer and stroke nurse follow-up (n=14). Follow-up was by telephone at 1, 4 and 9 weeks. Outcome measures were assessed after 12 weeks. Participants’ views on the intervention and research methods were explored using content analysis of poststudy focus group and interview data.

## What did I find?

My first SR^4^ identified four eligible studies. While individual studies reported increased aerobic capacity, meta-analysis found no significant change in any cardiovascular risk factors. Thus, evidence of the effectiveness of early post-TIA secondary prevention lifestyle interventions was limited. My second SR^5^ included 11 studies of home-based CR with good methodological quality and identified the use of 20 different BCTs. The most frequently used were social support (unspecified) (11 studies) and goal setting (behaviour) (10 studies).

In the feasibility study,^6^ 28 patients were invited to participate: 15 (10 men, 5 women; 9 TIA, 6 minor stroke; mean age 69 years) consented and completed all assessment measures except VO_2max_ testing, which all declined. The intervention was welcomed, and pedometers were valued highly, particularly for goal setting.

In the pilot study, 35.2% of eligible patients (44/125) consented to contact from a researcher; 90.9% of these (40/44) participated and 97.5% (39/40) completed the study. At 12-week review, cardiovascular risk factors in both intervention arms had improved. Qualitative data confirmed the feasibility and acceptability of the research methods and intervention.

## What is the most important clinical impact/practical application

The study’s recruitment and retention rates, and the intervention’s acceptability and potential effects, indicate that an RCT of a novel home-based CR programme based on ‘*The Healthy Brain Rehabilitation Manual*’,^6^ implemented early after a first TIA/minor stroke, is feasible, with important impact on secondary prevention of stroke.

**Figure 1:**
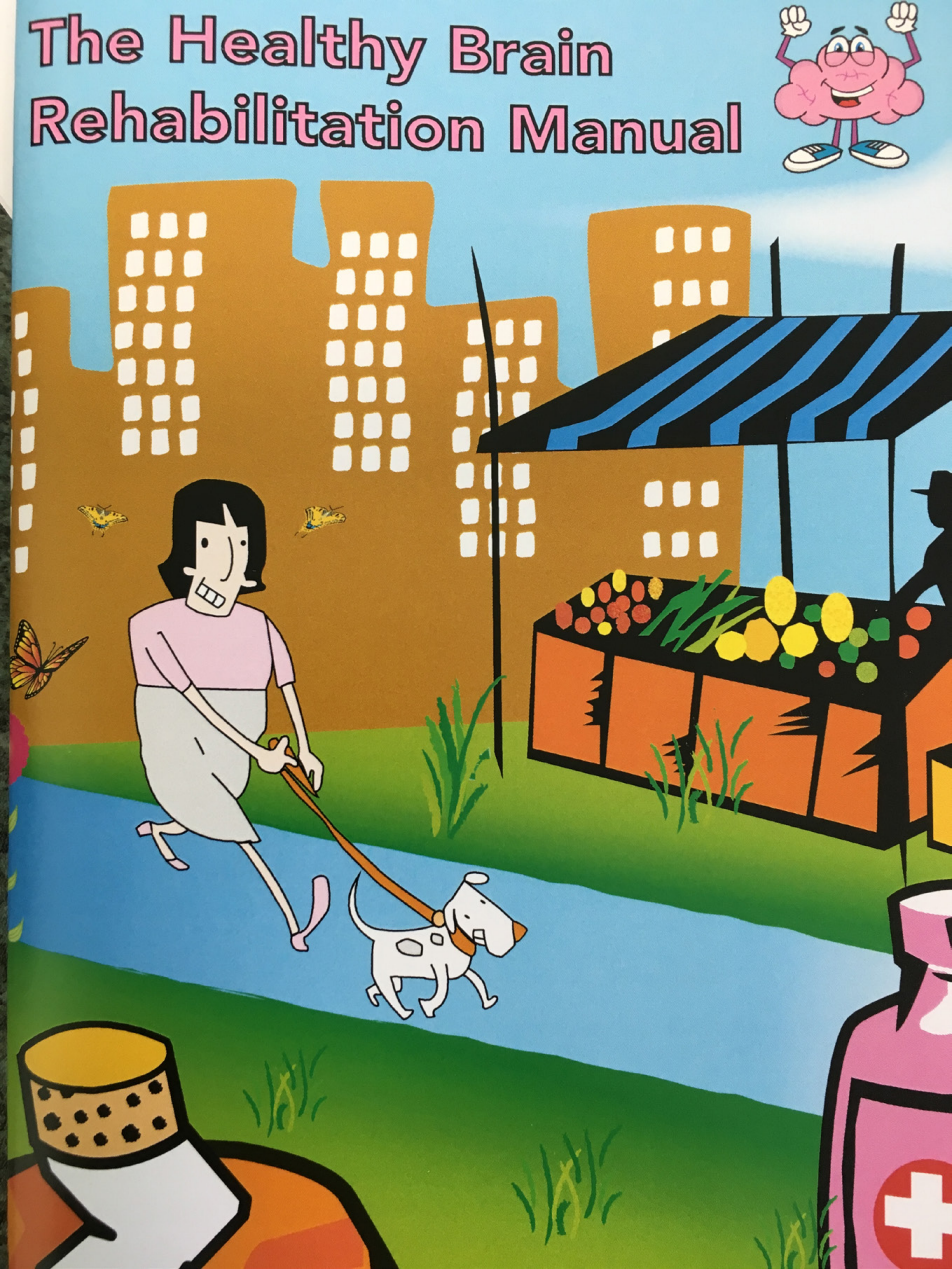
"The Healthy Brain Rehabilitation Manual" developed through the PhD study.
